# Effect of screw pressing temperature on yield, bioactive compounds, and quality of hemp (*Cannabis sativa* L.) seed oil

**DOI:** 10.1186/s42238-025-00296-6

**Published:** 2025-06-18

**Authors:** Aymane Allay, Abdessamad Ben Moumen, Youssef Rbah, Marie-Laure Fauconnier, Jacques Nkengurutse, Hana Serghini Caid, Ahmed Elamrani, Farid Mansouri

**Affiliations:** 1 Laboratory of Agricultural Production Improvement, Biotechnology, and Environment, Faculty of Sciences, Mohammed I University, Oujda , B.P. 717, 60000, Morocco; 2 Laboratory of Chemistry of Natural Molecules, Gembloux Agro-Bio Tech, University of Liège, Passage des Déportés, 2, 5030, Gembloux, Belgium; 3 Biology Department, Faculty of Sciences, University of Burundi, P.O. Box 2700, Bujumbura, Burundi; 4 Higher School of Education and Training, Mohammed I University, BP-410, Oujda, 60000 Morocco

**Keywords:** Hemp seed oil, Screw press, Temperature effect, Bioactive compounds, Pigments, Oxidative stability

## Abstract

**Background:**

Screw press extraction is widely used due to its simplicity and efficiency, but the impact of extraction temperature on oil characteristics requires further study. Existing studies highlight that high temperatures can improve yield but compromise oil quality. This study aims to assess the effects of different screw press extraction temperatures on hemp oil yield, composition and quality.

**Methods:**

The study was conducted to examine the influence of screw press extraction temperatures (30, 60, 80, 100, 120 and 140 °C) on hemp oil yield and quality. Extracted oils were analyzed according to key parameters, including oil yield, total phenolic content, tocopherols, oxidation stability index (OSI), pigments, quality indices (free fatty acids (FFA), peroxide value (PV), conjugated diene (K232) and conjugated triene (K270)), fatty acid profiles and color parameters (L*, a*, b*, C*_ab_ and h_ab_). The differences between oils extracted by pressing at different temperatures were evaluated via one-way multivariate analysis of variance.

**Results:**

The temperature played a key role in yield, reaching a maximum of 21.82%, representing an oil recovery of 66.68% at 100 °C, an improvement of approximately 4% over cold pressing (30 °C). Increasing the extraction temperature from 30 °C to 140 °C resulted in an increase in tocopherol content from 410.84 mg/kg to 512.98 mg/kg. On the other hand, temperature had no significant effect on the TPC, which varied from 31.76 mg GAE/kg to 41.89 mg GAE/kg as confirmed by HPLC-DAD/ESI-MS² analysis. On the other hand, oils extracted at higher temperatures were lower in quality, with higher PV, ranging from 6.36 meq O_2_/kg at 30 °C to 13.86 meq O_2_/kg at 140 °C, accompanied by a slight decrease in OSI. In addition, a decrease in chlorophyll-a content was observed, from 54.60 mg/kg at 30 °C to 36.10 mg/kg at 140 °C, which also led to a reduction in the a* value (green hue). Despite these alterations, the fatty acid profiles remained constant, whatever the thermal regime applied.

**Conclusion:**

Extraction temperature has a significant influence on the yield and quality of hemp oil. Underline the importance of selecting the optimum extraction temperature to balance yield and quality.

## Introduction

Hemp seed (*Cannabis sativa* L.) is recognized as a highly nutritious and versatile food source, attracting growing interest from the food and nutraceutical industries. Its composition includes 25–35% lipids with an optimal balance of essential fatty acids, 20–25% high-quality protein, and 20–30% carbohydrates, primarily in the form of dietary fiber. It is also a valuable source of vitamins (E, B1, B6) and essential minerals such as magnesium, iron, and zinc, further enhancing its appeal as a functional food with potential health benefits (Farinon et al. 2020; Irakli et al. 2019; Oseyko et al. 2019).

Hemp oil, extracted from the seeds, stands out for both its nutritional and environmental benefits. Its production aligns with a circular economy approach, ensuring that all parts of the seed are utilized efficiently. The oil extraction process generates a protein and fiber rich meal, which is a valuable by-product for the food and nutraceutical industries (Farinon et al. 2020; Visković et al., 2023). Hemp proteins are highly digestible and contain a well-balanced composition of essential amino acids, particularly arginine, which is beneficial for cardiovascular health (Irakli et al. 2019). These proteins are increasingly used in plant-based protein powders, functional foods, and meat substitutes, while their emulsifying and texturizing properties make them useful in various food formulations (Zhang et al. 2025). Additionally, the fiber content in hemp meal, primarily insoluble fiber, contributes to improved digestive health and lipid metabolism regulation. These fibers can be incorporated into fiber-enriched food products like energy bars and cereals, or even utilized in biomaterials and biodegradable packaging, supporting sustainability efforts (Burton et al. 2022; Taaifi et al. 2021).

In recent years, scientific research on hemp seed oil has intensified, primarily due to its exceptional lipid profile. It is rich in unsaturated fatty acids, particularly linoleic (omega-6) and α-linolenic (omega-3) acids, with an ideal 3:1 ratio that offers various health benefits (Taaifi et al. 2021). These fatty acids contribute to anti-inflammatory effects, improved skin hydration, and reduced dependence on topical treatments for dryness-related conditions. Moreover, hemp oil is an excellent source of bioactive compounds, including tocopherols, phytosterols, polyphenols, and carotenoids, which not only provide antioxidant properties but also protect the unsaturated fatty acids from oxidative degradation, extending the oil’s shelf life and enhancing its functional value (Farinon et al. 2020; Irakli et al. 2019).

Hemp seed oil is generally extracted via mechanical cold-pressing methods to preserve oil quality (Da Porto et al. 2012; Farinon et al. 2020). Compared with other extraction techniques, mechanical pressing is preferred for health, safety, economic and environmental reasons (Nde & Anuanwen, 2020). This process results in the production of oil that is well suited for human consumption and for use in various body oils and creams because of its excellent skin absorption properties (Callaway 2004). In addition, the method enables the utilization of residual meals, has relatively low initial and operational costs, and produces oil that is free from solvent residues, ensuring a more natural and unaltered final product (Nde & Anuanwen, 2020).

Cold pressing involves the use of a screw or hydraulic press. During cold pressing, hemp seeds are mechanically pressed at low temperature, which preserves the natural components of the oil, such as polyunsaturated fatty acids (PUFAs), vitamins and antioxidants, notably tocopherols and polyphenols (Occhiuto et al. 2022). In addition, cold-pressed hemp seed oil is known for its distinct greenish color, which stems from the chlorophyll present in the seeds and its nutty taste, making it popular for culinary use as well as in health supplements and cosmetics (Farinon et al. 2020).

However, the efficiency of current mechanical oil extraction equipment and processes is often considered insufficient (Ahangari et al. 2021). As a result, the yield of mechanical pressing is generally lower than that obtained by solvent extraction methods. Although solvent extraction results in relatively high yields, it often compromises oil quality, which is unacceptable for high-value oils. In addition, mechanical extraction is considered an environmentally friendly method, as it does not use chemical extraction solvents such as hexane and does not require excessive energy input, making it a sustainable choice for oil production. As a result, the production process for these oils remains limited to mechanical extraction methods.

Owing to these advantages of press-extracted hemp seed oil, researchers have turned their attention to optimizing extraction processes to maximize yield. Among these works, Aladić et al. (2014) conducted an in-depth study to assess the impact of three key parameters, namely, the pressure nozzle size, extraction temperature and rotation frequency. These factors play crucial roles in extraction efficiency and directly influence the volume of oil recovered. In addition, Golimowski et al. (2022) and Crimaldi et al. (2017) studied the effect of extraction temperature on yield. However, despite efforts to improve yield, significant gaps remain in the scientific literature. To the author’s knowledge, no detailed study has explored the impact of the high temperatures used during pressing on the chemical composition of the extracted oil. Crucial aspects, such as the effect of temperature on bioactive compounds (such as polyphenols and tocopherols), pigments (chlorophylls and carotenoids), and other quality markers, have not been well studied. Understanding these interactions is essential to ensure that yield optimization is not achieved at the expense of the nutritional properties of hemp seed oil.

This research aimed to (i) analyze the effect of extraction temperature, via a screw press, on the yield and quality of hemp seed oil; (ii) assess the impact of temperature on the various antioxidants present in the oil, their concentration and their contribution to oxidative stability; and (iii) compare oils obtained by pressing at different temperatures with those extracted via the Soxhlet method using *n*-hexane as the solvent. As a result, we hypothesized that higher temperatures may increase oil yields and thus improve extraction. On the other hand, higher temperatures may affect valuable compounds such as antioxidants and fatty acid composition. To this end, six temperatures (30, 60, 80, 100, 120 and 140 °C) were used to extract oil from hemp seeds via a screw press, followed by a comprehensive evaluation to examine the influence of extraction temperature on oil yield, total phenolic content, tocopherols, oxidation stability, pigments (total carotenoids and chlorophyll a and b), quality indices (percentages of free fatty acids (%FFA), peroxide value (PV), conjugated diene (K232) and conjugated triene (K270)), fatty acid profiles and color parameters (L*, a*, b*, C*_ab_, h_ab_).

## Materials and methods

### Chemicals and reagents

The standards, comprising tocopherols (α-tocopherol, β-tocopherol, γ-tocopherol and δ-tocopherol), gallic acid, and fatty acid methyl esters (FAME), were obtained from Sigma‒Aldrich (St. Louis, MO, USA). Analytical-grade *n*-hexane was used as the extraction solvent, while all other chemicals and reagents used in this study were of analytical grade and sourced from Merck Chemical Company (Darmstadt, Germany).

### Plant material

The experimental trials carried out in this study used hemp (*Cannabis sativa* L.) seeds of the ‘Beldia’ variety grown in the Jebha region of northern Morocco. The seeds were supplied by the *Agence Nationale des Plantes Médicinales et Aromatiques* (ANPMA), Morocco. After being harvested, the seeds were stored in plastic bags at a temperature of 2–4 °C in a refrigerator until use. The moisture content was determined to be 5.13 ± 0.10% via the standard hot-air oven method at 105 ± 1.00 °C until a constant weight was obtained (Mansouri et al. 2023).

### Hemp seed oil extraction

#### Solvent extraction

Soxhlet extraction was accomplished according to Allay et al. (2024b). Approximately 30 g of crushed seeds were weighed and placed in a cellulose cartridge. The cartridge was then placed in the extraction chamber of a 250 mL Soxhlet apparatus, fitted with a condenser and connected to a 250 mL distillation flask containing 200 mL of *n*-hexane. The seed oil was extracted by refluxing with *n*-hexane for 6 h. The hexane was then removed under vacuum using a rotary evaporator heated to 40 °C. The oil was then stored under a nitrogen atmosphere to prevent oxidation and then kept in a freezer at -18 °C for further analysis. All extraction processes were carried out in triplicate, and the mean values were recorded. The yield of the oily extracts was expressed via Eq. (1):

1 $$\:Oil\:yield\:\left(\%\right)=\frac{\left(mass\:of\:soxhlet\:oil\right)\:}{\left(mass\:of\:seed\:powder\right)}\:x\:100$$.

#### Mechanical extraction (Single-screw extrusion)

Hemp seed oil extraction was carried out via a single-screw extruder specifically designed for the process. The unit was equipped with a 1.7 kW motor operating at a maximum voltage of 240 V and a maximum current of 4.5 A with a capacity of 700 g/h. The extruder’s technical specifications included a 20 cm-long screw with a 2 cm pitch, an internal diameter of 1.20 cm, and a channel depth of 0.50 cm. The sleeve had an internal diameter of 2.5 cm and was equipped with an outlet filter perforated at its end to separate the extracted oil. The filter perforations had a diameter of 2 mm, enabling efficient oil separation. To ensure uniform extraction, screw rotation speed was kept constant at 32 rpm.

A temperature-controlled heating ring was used to preheat the press head to the target temperature. Preheating lasted between 10 and 15 min before the start of each experiment, ensuring thermal stability. Once stable operating conditions were reached, 100 g of hemp seeds were introduced into the hopper and pressed at six different temperatures (30, 60, 80, 100, 120 and 140 °C). The extraction time recorded for all samples was approximately 10 min. Each condition was tested in triplicate to ensure reproducible results.

The crude oil obtained after pressing was centrifuged at 7000 × g for 15 min to remove residual fine particles, producing a clarified oil of higher quality. This clarified oil was then stored under a nitrogen atmosphere to prevent oxidation and then kept in a freezer at -18 °C for further analysis. To assess the efficiency of the extraction process, three key parameters were calculated and expressed in grams per 100 g of seed (%). These parameters quantify extraction performance in terms of material recovery and losses:

-TM (Total Mass): This is the percentage of the total mass recovered after extraction, which includes the extracted oil plus the sediment. This parameter is used to evaluate the total amount of material recovered after the extraction process.

2 $$\:TM\:\left(\%\right)=\frac{\left(summation\:of\:mass\:produsts\right)\:}{\left(mass\:of\:hemp\:seed\:samples\right)}\:x\:100$$

-MML (Mass of Material Losses): This percentage represents material losses after extraction, including oil lost after centrifugation plus sediment. In other words, it reflects the amount of material that could not be collected after separation.

3 $$\:MML\:\left(\%\right)=\frac{\left(\text{o}\text{i}\text{l}\:\text{l}\text{o}\text{s}\text{t}\:\text{a}\text{f}\text{t}\text{e}\text{r}\:\text{c}\text{e}\text{n}\text{t}\text{r}\text{i}\text{f}\text{u}\text{g}\text{a}\text{t}\text{i}\text{o}\text{n}\:\text{p}\text{l}\text{u}\text{s}\:\text{s}\text{e}\text{d}\text{i}\text{m}\text{e}\text{n}\text{t}\right)\:}{\left(mass\:of\:hemp\:seed\:samples\right)}\:x\:100$$.

-Oil yield (OY): according to the mass of oil after centrifugation i.e. without sediment.

4 $$\:OY\:\left(\%\right)=\frac{\left(clear\:oil\:after\:separation\:\right)\:}{\left(mass\:of\:hemp\:seed\:samples\right)}\:x\:100$$.

The yield of the oil extracted by the mechanical method was expressed as the content of oil extracted by the Soxhlet method via the following Eq. (5):

5 $$\eqalign{& \>Oil\>recovery\>\left( \% \right) \cr & = {{\left( {Yield\>of\>oil\>extracted\>by\>mechanical\>method} \right)\>} \over {\left( {Yield\>of\>oil\>extracted\>by\>Soxhlet\>method} \right)}}\>x\>100 \cr} $$.

### Physicochemical characterization of the extracted oils

#### Tocopherol analysis

The analysis of tocopherols as described by Ben Moumen et al. (2015) was carried out by high-performance liquid chromatography (HPLC) equipped with a diode array detector (DAD). Separation of α-, β-, γ- and δ-tocopherols was performed on a silica-based Uptisphere 120Ǻ NH_2_ column (150 mm × 3 mm, 5 μm particle size) with a mobile phase consisting of *n*-hexane/2-propanol (99:1, v/v) at a flow rate of 1 mL/minute. Detection was performed at 292, 296 and 298 nm for tocopherol quantification. The tocopherol content of the oil samples was identified by comparing their retention times and peak areas with those of commercial tocopherol standards, and the results were expressed as milligrams of tocopherol per kilogram of oil (mg/kg).

#### Total phenolic content and HPLC-DAD/ESI-MS^2^ analysis

The total phenolic content (TPC) was determined via the method described by Allay et al. (2024a). The oil samples were subjected to liquid‒liquid extraction via an 80/20 (v/v) methanol/water mixture to extract phenolic compounds. The extracts were then reacted with Folin-Ciocalteu reagent, which, in the presence of phenolic compounds, produces a blue color. After sodium carbonate was added to the mixture and incubated in the dark for 90 min, the absorbance was measured at 765 nm via a spectrophotometer. The TPC was quantified using a gallic acid standard curve, and the results were expressed in milligrams of gallic acid equivalents (GAE) per kilogram of oil.

Analysis of phenolic compounds was carried out via the use of methanolic extracts of extracted oil at different temperatures, which were injected into an Agilent 1260 Infinity II high-performance liquid chromatography system (Agilent Technologies, USA) following the protocol described in previous studies (Benkirane et al. 2022; Allay et al. 2024a). The separation of phenolic compounds was performed on a C18 column (120 × 4.6 mm, 3.5 μm particles) and monitored at several specific wavelengths (254, 280, 300 and 340 nm). After separation, the compounds were analyzed using a Bruker Esquire HCT mass spectrometer (Bruker, Germany).

#### Total chlorophyll and carotenoid contents

Analysis of total chlorophyll and carotenoid contents was carried out according to the procedure described by Aladić et al. (2014). A total of 0.10 g of the oil sample was dissolved in 5 mL of diethyl ether. The mixture was thoroughly mixed and then subjected to ultrasonic extraction for one minute to ensure efficient pigment extraction. After extraction, the absorbance of the solution was measured at wavelengths of 470 nm for carotenoids and 640 nm and 663 nm for chlorophylls, using a UV‒visible spectrophotometer. The total chlorophyll and carotenoid concentrations were calculated using specific equations that correlate absorbance readings with pigment concentrations, and the results are expressed in milligrams per kilogram of oil (mg/kg).

6 $$\eqalign{& Chlorophyll\;a\;\left( {mg/kg} \right)\; \cr & {\rm{ = \;}}{{\left( {9,93\; \times \;{A_{663\;}}} \right)\, - \left( {0,78\; \times \;{A_{640}}} \right)} \over W} \cr} $$

7 $$\eqalign{& Chlorophyll\;b\;\left( {mg/kg} \right)\; \cr & {\rm{ = \;}}{{\left( {17,60\; \times \;{A_{640\;}}} \right)\, - \,\left( {2,81 \times \;{A_{663}}} \right)} \over W} \cr} $$

8 $$\eqalign{& Total\;Carotenes\;\left( {mg/kg} \right)\; \cr & {\rm{ = }}\;{{\left( {1000\; \times \;{A_{470\;}} - 0.52 \times Chly\;a\; - 7.25\; \times \;Chly\;b} \right)} \over {226\; \times \;W}} \cr} $$

where A is the absorbance at the specified wavelengths (663, 640, and 470 nm) and W is the weight of hemp seed oil.

#### Colorimetric analysis

The color of the extracted oils was objectively assessed using CIELAB parameters (L*, a*, b*), which are commonly used to characterize colorimetric properties. The L* parameter measures sample brightness, whereas the a* and b* coordinates indicate chromatic positions on the green–red and blue–yellow axes, respectively. After careful homogenization of the samples, colorimetric measurements were carried out directly using a KONICA MINOLTA Chroma Meter CR-410 (measuring area ⌀ 50 mm).

Cylindrical coordinates C*_ab_ and h_ab_ are calculated from a* and b* using Eqs. (9) and (10):

9 $$\:{C*}_{ab}\text{=}\sqrt{{a}^{*2}+{b}^{*2}}$$

10 $$\:{h}_{ab}\text{=}{\:(\text{t}\text{a}\text{n}}^{-1}\left(\frac{b*}{a*}\right)\:\times\:\:57.29)\:+\:180$$

#### Fatty acid analysis

The fatty acids in the oil samples were analyzed via gas chromatography mass spectrometry (GC‒MS). Prior to injection, the extracted triglycerides were first derivatized to the corresponding methyl esters via the 1 K-07 AOCS 2007 method described by Allay et al. (2024b). FAME were then injected into a GC‒MS 1300/TS Q 8000 Evo THERMO system equipped with a TR-5 capillary column (30 m long, 0.25 mm internal diameter and 0.25 μm film thickness) with 5% phenyl methyl polysiloxane. The injection was carried out at 250 °C in split mode, with an injection volume of 1 µL. The oven temperature was initially set at 120 °C for 1 min, then increased to 180 °C at 3 °C/minute and held for 15 min, followed by an increase to 240 °C at 5 °C/minute and held for 25 min. The carrier gas used was helium at a flow rate of 1 mL/minute. After separation, the FAME were introduced into the quadrupole mass spectrometer (the ionization details were well explained elsewhere by Occhiuto et al. (2022). Each sample was analyzed in triplicate, and FAME peaks were identified based on mass spectra from the NIST database, those reported in the literature and retention times of a standard 37-component FAME mixture (Sigma Aldrich). The results are expressed as a percentage of total fatty acids.

#### Oxidative stability index

The oxidation stability index (OSI) was determined following the method described by Mansouri et al. (2023). A 3.00 ± 0.01 g portion of the oil sample was subjected to accelerated oxidation using a Rancimat instrument, under a constant temperature of 100 °C and an air flow rate of 20 L/h. The OSI value, expressed in hours, corresponds to the induction period, i.e., the time required for the formation of volatile oxidation products that cause a marked increase in the conductivity of the distilled water. This parameter serves as a reliable indicator of the oil’s oxidative stability and its resistance to thermal degradation.

#### Oil quality indices

Key parameters such as free fatty acids (FFA), the peroxide value (PV) and UV absorbance at 232 and 270 nm were evaluated. These indices provide a comprehensive assessment of oil quality, freshness and oxidation state.

-FFA, analysis is carried out by potassium hydroxide titration in the presence of phenolphthalein, according to the official European method for virgin olive oil EEC/2568/91 (Mansouri et al. 2019). The results were expressed as a percentage of linoleic acid using the Eq. (11):

11 $$\:\text{\%}\text{F}\text{F}\text{A}\:\text{a}\text{s}\:\text{l}\text{i}\text{n}\text{o}\text{l}\text{e}\text{i}\text{c}\:\text{a}\text{c}\text{i}\text{d}\:=\:\frac{\text{V}\:\times\:\:\text{N}\:\times\:\:28.05}{\text{P}\text{E}}$$.

N: Normality of potassium hydroxide (0.01 N).

V: Volume of KOH poured, expressed in mL.

PE: Test sample in grams.

-PV, this is determined by titration after reaction with potassium iodide according to EEC/2568/91 (Mansouri et al. 2019). The result is expressed in milliequivalents of oxygen per kg of oil (meq O₂/kg), using the Eq. (12):

12 $$\:\text{P}\text{V}\:(\text{m}\text{e}\text{q}\:{\text{O}}_{2}/\text{K}\text{g})=\:\frac{(\text{V}\text{E}\:-\:\text{V}\text{B})\:\times\:\:0.01\:\times\:\:1000}{\text{P}\text{E}}$$.

N: Normality of sodium thiosulphate (0.01 N).

VE: Volume in mL of sodium thiosulfate added during titration.

VB: Volume in mL of sodium thiosulfate used for the blank.

PE: Test sample in grams.

-Specific extinction coefficients (K232 and K270), measured by UV spectrophotometry at 232 nm and 270 nm, they are calculated from the optical density of a 1% solution of oil in cyclohexane, according to EEC/2568/91 (Mansouri et al. 2019). Specific extinction values are calculated according to the following Eq. (13):

13 $$\:\text{K}{\uplambda\:}=\:\frac{\text{A}{\uplambda\:}}{\text{C}\:\times\:\text{L}}$$.

Kλ: specific extinction at wavelength λ.

Aλ: absorbance measured at wavelength λ.

C: solution concentration in grams per 100 ml.

L: bowl thickness in centimeters.

#### Statistical analysis

The differences between oils extracted by pressing at different temperatures were evaluated via one-way multivariate analysis of variance (MANOVA) with SPSS software, version 25. A *p* value of less than 0.05 was considered statistically significant. Before performing the ANOVA, the normality of the data was checked via the Shapiro Wilk test, which confirmed that the data followed a normal distribution (*p* > 0.05). In addition, the homogeneity of variances was tested via Levene’s test, which confirmed that the variances were homogeneous (*p* > 0.05). In addition, a principal component analysis (PCA) was performed. Before performing the PCA, data adequacy was assessed via the Kaiser Meyer Olkin (KMO) test and Bartlett’s test (*p* < 0.05). Finally, the data obtained were analyzed via Prism 8.0 (GraphPad) software, and the results are presented as the means ± standard deviations.

## Results and discussion

To evaluate the effect of temperature on the yield, composition and quality of hemp seed oil extracted via a screw press, six temperatures (30, 60, 80, 100, 120 and 140 °C) were used. In addition, throughout the experiment, the screw rotation speed and the diameter of the restriction matrix remained constant. The results of these experiments, including the oil yield, TPC, tocopherol content, oxidative stability index (OSI), chlorophyll a and b content, total carotenoid content, quality index (% FFA, PV and extinction coefficients), fatty acid profile and color (L*, a*, b*, C*_ab_, h_ab_), are presented in Tables 1, 2, 3, 4 and 5.

**Table 1:** Results of oil yield of temperature oil, total mass (TM), mass of material losses (MML), oil yield (OY), and oil recovery obtained during extraction of hemp seeds oil using a screw press at different temperatures

Temperature of extraction (°C)	Oil temperature (°C)	TM (%)	MML (%)	OY (%)	Oil recovery (%)
Cold pressing (30)	28.05 ± 0.05a	22.90 ± 0.24a	5.09 ± 0.06a	17.80 ± 0.19a	54.41 ± 0.57a
60	42.57 ± 0.51b	24.12 ± 0.20b	5.70 ± 0.12ab	18.42 ± 0.28a	56.31 ± 0.85a
80	46.80 ± 0.88c	27.15 ± 0.33c	5.44 ± 0.47ab	20.33 ± 0.24b	62.12 ± 0.73b
100	50.53 ± 1.44d	27.92 ± 0.33c	6.17 ± 0.63b	21.82 ± 0.15c	66.68 ± 0.42c
120	57.45 ± 1.29e	27.81 ± 0.36c	7.59 ± 0.10c	21.64 ± 0.34c	66.13 ± 1.04c
140	62.22 ± 1.08f	28.74 ± 0.29d	7.46 ± 0.10c	21.71 ± 0.39c	66.35 ± 1.20c
Soxhlet (*n*-hexane)	––	––	––	32.72 ± 0.37d	100

Mean ± SD of three independent experiments (*n* = 3). abcd different letters indicate significant differences (*p* < 0.05) within columns

TM: percentage of total mass (extracted oil + sediment)

OY: Oil yield is expressed in g per 100 g of seeds (%) according to the mass of oil recovered after centrifugation i.e. without sediment

MML: percentage of mass of material losses (represents material losses after extraction, including oil lost after centrifugation and sediment)

Oil recovery is expressed as a percentage of the yield of oil extracted by screw press over the yield of oil extracted by Soxhlet

### Influence of extraction temperature on oil yield

**Fig. 1:**
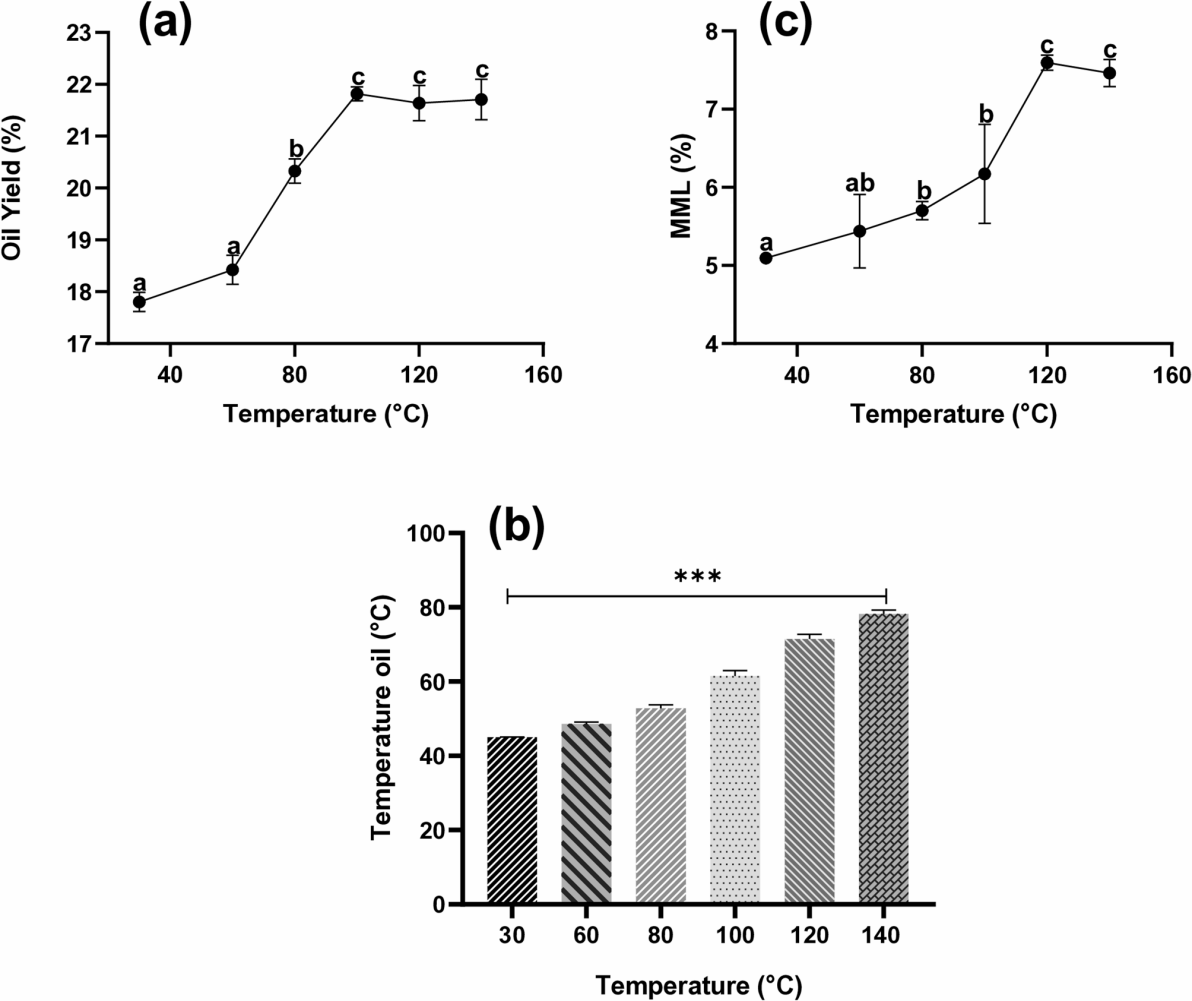
Influence of pressing at different temperatures on (**a**) oil yield, (**b**) temperature oil, and (**c**) mass of material losses (including oil lost after centrifugation and sediment) (MML) of hemp seed oils extracted by using a screw press. Bars with the same letter are not significantly different from other samples of the same fraction, *** significant with *p*<0.05

The variations in oil yield (OY), total mass percentage (TM), mass loss percentage (MML), oil temperature and oil recovery rate in response to different hemp seed extraction temperatures are presented in Table 1; Figs. 1a-c.

A maximum extraction of 21.82%, corresponding to 66.68% of the total oil available, was obtained at an extraction temperature of 100 °C, while the lowest yield of 17.80% (or 54.41% of the total oil) was observed at 30 °C. Our results were consistent with those reported by Crimaldi et al. (2017) indicating oil recoveries between 58.40 and 73.38% of hemp seed oil extracted by screw pressing at different conditions. On the other hand, Golimowski et al. (2022) observed oil recoveries between 67.00 and 90.60% for different hemp seed varieties under different conditions, which were much greater than those obtained in this study. These variations could be attributed to factors such as genotype, the configurations of the pressing equipment used differ, and the modalities of the established parameters (screw rotation speed, restriction diameter, and seed preparation) also vary. Of course, it was difficult to compare all the data with each other. Nevertheless, it was possible to assess overall trends in the effect of extraction temperature on OY.

Overall, and as expected, the increasing extraction temperature had a significant (*p* < 0.05) impact on improving OY. As shown in Fig. 1a, OY initially increased in proportion to the rise in temperature, from 30 to 100 °C. Above this value, OY reached a plateau, indicating that a further rise in temperature no longer resulted in a significant (*p* < 0.05) improvement in OY. Interestingly, a relatively moderate temperature increase of just 30 °C (from 30 °C to 60 °C) already resulted in a significant (*p* < 0.05) improvement in OY. This effect was attributed to the heat-induced reduction in oil viscosity, thus facilitating its flow and the movement of residual cake into the expeller (Muangrat et al. 2018).

These results are in line with previous studies, including those by Aladić et al. (2014), Golimowski et al. (2022) and Crimaldi et al. (2017), all of whom have highlighted a significant increase in the OY of hemp seeds with temperature. Specifically, these researchers observed an increase in yield between 60 and 100 °C, 20 and 60 °C, and 50 and 70 °C. Moreover, they emphasized that this trend was independent of other extraction parameters, such as nozzle diameter or screw rotation frequency.

Furthermore, Fig. 1b shows that different extraction temperatures had a significant (*p* < 0.05) effect on the oil outlet temperature. Increasing the temperature from 30 to 140 °C increased the oil temperature from 28.05 to 62.22 °C. Our results were consistent with those reported by Aladić et al. (2014), indicating that increasing the extraction temperature from 60 to 100 °C increased the hemp seed oil temperature from 41 to 49 °C. However, this increase was not entirely decisive. Increasing the temperature from 30 to 160 °C only slightly increased the hemp oil temperature by approximately 34 °C, which was not proportional to the applied temperature increase. Similarly, Aladić et al. (2014) reported an increase of only 8 °C in hemp seed oil temperature when extraction temperatures ranging from 60 to 100 °C were used. This phenomenon was observed in different oils. Furthermore, in the case of almond oil and pistachio oil, researchers have concluded that the applied temperature does not directly affect the oil itself (Rabadán et al. 2018).

However, although increasing the temperature had a positive effect on the OY, it also had negative effects. As shown in Fig. 1c, increasing the extraction temperature from 30 to 140 °C increased the percentage of MML from 5.09 to 7.59%. These results are in agreement with the findings of Aladić et al. (2014), who reported that increasing the extraction temperature from 60 to 100 °C led to an increase in the percentage of insoluble impurities in hemp seed oil extracted via a screw press. In addition, Muangrat et al. (2018) reported that higher temperatures increased the proportion of solid residues and sediments present in oil extracted from *Sacha Inchi* seeds. This highlights the need for an additional filtration step to obtain high-purity oil.

### Influence of the extraction temperature on the physicochemical parameters of the oil

Despite the positive effect of temperature on OY, high temperatures can have a significant effect on the quality and composition of the extracted oil. It was therefore essential to analyze and characterize oils obtained at different extraction temperatures via a screw press. For this study, only purified oils, free of cake sediment, were evaluated, considering various physicochemical and compositional parameters, to better understand the influence of extraction temperature on their properties.

**Table 2:** Results of total phenolic content (TPC), tocopherols, chlorophyll a-b, total chlorophyll, and carotenoid of hemp seed oil extracted using a screw press at different temperatures

Temperature of extraction (°C)	TPC	Tocopherols				Chlorophyll a	Chlorophyll b	Total Chlorophyll	Total carotenoid
		Alpha	Gama	Delta	Total				
Cold pressing (30)	31.76 ± 1.18b	34.38 ± 0.48c	348.02 ± 8.42a	28.44 ± 0.10b	410.84 ± 7.92a	54.60 ± 1.80d	29.40 ± 1.60b	84.00 ± 8.40d	24.10 ± 0.20b
60	32.82 ± 0.98b	34.01 ± 1.78c	355.22 ± 3.05a	28.07 ± 1.26b	417.30 ± 4.25a	52.30 ± 1.10 cd	28.40 ± 1.40b	80.80 ± 3.30d	25.30 ± 0.30bc
80	37.08 ± 0.66c	36.73 ± 0.31c	417.34 ± 9.90b	30.9 ± 0.64c	484.97 ± 9.16b	49.40 ± 1.70c	28.60 ± 1.50b	78.80 ± 4.30 cd	26.50 ± 0.80bc
100	37.52 ± 0.87c	34.28 ± 0.04c	402.80 ± 27.60b	29.17 ± 0.16bc	466.25 ± 27.69b	49.00 ± 2.30c	27.90 ± 0.60b	76.90 ± 1.30c	29.60 ± 0.40 cd
120	41.89 ± 1.31d	35.32 ± 0.42c	401.67 ± 5.57b	29.28 ± 0.07bc	466.27 ± 5.86b	38.20 ± 1.40b	28.10 ± 1.20b	66.30 ± 2.80b	31.60 ± 0.40d
140	38.29 ± 0.77c	28.43 ± 0.85b	454.93 ± 2.12c	29.62 ± 1.30bc	512.98 ± 4.83c	36.10 ± 2.40b	27.20 ± 0.80b	63.30 ± 3.20b	31.40 ± 0.20d
Soxhlet (*n*-hexane)	19.43 ± 1.13a	21.66 ± 0.93a	377.74 ± 11.03a	9.57 ± 0.94a	408.98 ± 11.02a	4.80 ± 0.65a	3.23 ± 0.15a	8.03 ± 0.50a	1.78 ± 0.03a

Mean ± SD of three independent experiments (*n* = 3). abcd different letters indicate significant differences (*p* < 0.05) within columns

Total phenolic content (TPC) is expressed in mg gallic acid equivalent per kg of oil (mg GAE/kg oil)

Tocopherols, chlorophyll a, b and total carotenoid are expressed in mg per kg of oil (mg/kg oil)

#### Tocopherols

Tocopherol plays a key role in the oxidative stability, antioxidant properties and sensory characteristics of oil. The concentrations of different tocopherol isomers in hemp seed oil extracted by a screw press at various temperatures are presented in Table 2. The total tocopherol content ranged from 410.84 mg/kg at 30 °C to 512.98 mg/kg at 140 °C. Overall, the tocopherol contents in oils extracted by pressing at different temperatures were greater than those in the *n*-hexane-extracted oils. These results are in line with those reported by Farinon et al. (2020), who reported total tocopherol contents ranging from 143.30 to 929.70 mg/kg in cold-pressed hemp oils. However, some studies, such as that by Blasi et al. (2022), reported even higher concentrations, ranging from 655 to 1118 mg/kg. These variations could also be linked to genotype and growing conditions (Irakli et al. 2019). Among the isomers, γ-tocopherol was the most abundant, followed by the α and δ isomers, which is consistent with the observations of several studies (Allay et al. 2024a; Rezvankhah et al. 2018; Taaifi et al. 2021). Although α-tocopherol is considered the most potent antioxidant (Seppanen et al. 2010), γ-tocopherol plays a distinctive role in reducing lipoprotein oxidation and enhances the activity of superoxide dismutase, which plays a crucial role in free radical scavenging, often with greater efficiency than α-tocopherol (Farinon et al. 2020).

Table 2 highlights the significant (*p* < 0.05) impact of pressing temperature on the tocopherol content in hemp seed oil. As the temperature increases, the release of tocopherols particularly γ-tocopherol is enhanced, likely due to the breakdown of cellular structures, which facilitates their extraction. However, at 140 °C, a significant (*p* < 0.05) decline in α-tocopherol was observed, suggesting that it undergoes thermal degradation at elevated temperatures. This degradation phenomenon has been widely reported in other vegetable oils, such as sunflower and rapeseed oils, particularly during high-temperature deodorization processes (Kreps et al. 2017).

#### Total phenolic content

The TPC in hemp seed oil extracted by a screw press at various temperatures are presented in Table 2. The TPC ranged from 31.76 mg GAE/kg at 30 °C to 41.89 mg GAE/kg at 120 °C. Overall, the press-extracted oils had a higher TPC than did the *n*-hexane-extracted oils, which contained only 19.43 mg GAE/kg. In the literature, Farinon et al. (2020) reported TPC values for cold-pressed hemp oil ranging from 44 to 268 mg GAE/100 g, which are well above those obtained in this study. These variations could be attributed to factors such as genotype, environmental conditions and harvest year, which significantly influence the biosynthesis and accumulation of polyphenols in hemp seeds (Irakli et al. 2019). In addition, Faugno et al. (2019) reported that agricultural practices, harvesting and storage techniques, and pressing conditions also affect the phenolic profile of seeds.

Table 2 shows a slight increase in the TPC of the oil as the extraction temperature increased, reaching a maximum at 120 °C before decreasing at 140 °C. This moderate increase can be attributed to the release of phenolic compounds trapped in the cell structures, which is favored by the effect of heat on cell wall rupture. Similar observations were reported by Rabadán et al. (2018), who reported a significant increase in the TPC of walnut oils at high extraction temperatures. However, the decrease observed at 140 °C could be due to the thermal degradation of phenolic compounds. Basdogan et al. (2021) reported that the TPC of pumpkin seed oil decreased significantly at high temperatures, with a halving at 100 °C compared with cold extraction.

**Fig. 2:**
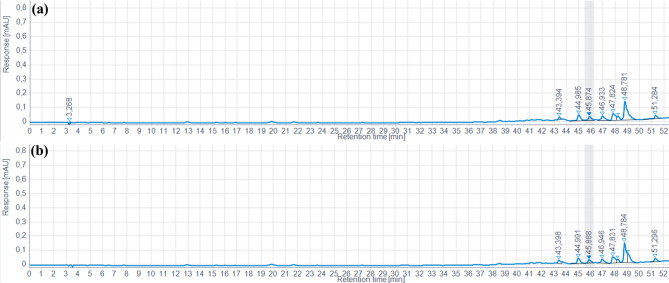
HPLC-DAD chromatogram recorded at 280 nm corresponding to hemp seed oil extract (*Cannabis sativa* L.), obtained by cold pressing (30 °C) (**a**) and pressing at 120 °C (**b**) using a screw press

Analysis of the chromatograms obtained by HPLC-DAD/ESI-MS^2^ enabled us to assess the phenolic compound profile in mechanically extracted oil at 30 °C (where TPC was low) and at 120 °C (where TPC was highest) (Fig. 2). The results clearly show that there is no change in the phenolic profile between the samples compared. Indeed, superposition of the chromatograms reveals a perfect match, with no new peaks appearing or disappearing. This suggests that temperature variations did not alter the phenolic composition of the oil, confirming the stability of these compounds under the conditions studied. These observations reinforce the idea that the extraction or treatment process did not induce any degradation or chemical transformation of the phenols present. However, although the phenolic profile remained stable, some detected peaks could not be identified, which could indicate the presence of unknown phenolic compounds or uncharacterized secondary metabolites.

Furthermore, the transfer of phenolic compounds from the seeds to the extracted oil proved limited. This limitation is mainly attributable to the hydrophilic nature of these compounds, which renders them poorly soluble in a lipid matrix such as oil. These results are consistent with the work of Maier et al. (2009), who demonstrated that, in the case of grape seed oil, TPC in the oil represents only 0.013–0.019% of the total polyphenols present in the seeds. This low content highlights the challenges associated with extracting phenolic compounds from oils, due to their affinity for aqueous rather than lipid phases.

#### Pigments and color

Pigments such as chlorophylls and carotenoids play essential roles in the visual, chemical and nutritional qualities of edible oils.

Unlike polyphenols and tocopherols, chlorophylls are powerful fat-soluble photosensitizers capable of inducing oxidation and consequently reducing shelf-life (Blasi et al. 2022; Izzo et al. 2020). The high content of total chlorophyll, represented essentially by two isomers, chlorophyll-a and chlorophyll-b, has a maximum absorbance at wavelengths of 640 nm and 663 nm, respectively. Table 2 shows the chlorophyll-a and chlorophyll-b contents of oils extracted via a screw press at different temperatures. The total chlorophyll content ranged from 63.30 to 87.80 mg/kg, with chlorophyll a and chlorophyll b concentrations ranging from 36.10 to 54.60 mg/kg and 27.20 to 29.40 mg/kg, respectively. Overall, the chlorophyll content of oils extracted by pressing was greater than that extracted by *n*-hexane, with 4.80 and 3.23 mg/kg chlorophyll a and b, respectively, for a total chlorophyll content of 8.03 mg/kg.

Blasi et al. (2022) reported a similar average content of 76.40 mg/kg, with 52.20 mg/kg chlorophyll-a and 24.30 mg/kg chlorophyll-b. In contrast, Izzo et al. (2020) reported very low total chlorophyll contents ranging from 0.41 to 2.64 mg/kg in hemp seed oil, but high variability was generally observed (Aachary et al. 2016; Liang et al. 2015). In the present study, the chlorophyll-a content was greater than the chlorophyll-b content, which was similar to what has been reported in other studies (Aachary et al. 2016; Blasi et al. 2022).

The carotenoid content was determined via spectrophotometric analysis via the maximum absorbance at a wavelength of 470 nm. In addition to tocopherols and phenols, carotenoids are considered powerful antioxidants that help protect oils from oxidative damage (Farinon et al. 2020). The total carotenoid content of the press-extracted oils ranged from 24.10 to 31.60 mg/kg. Overall, the press-extracted oils presented higher carotenoid contents than did the *n*-hexane-extracted oil (1.78 mg/kg). In addition, Blasi et al. (2022) reported lower values of 1.78 to 2.61 mg/kg in cold-pressed hemp oil. In contrast, Oomah et al. (2002) reported similar carotenoid concentrations (20.00 to 53.00 mg/kg) in mechanically extracted hempseed oil. Interestingly, chlorophyll content was around 2–3 times higher than carotenoid content, which was similar to the result obtained by Blasi et al. (2022), where the chlorophyll/carotenoid ratio was around 2.00-2.90.

Table 2 shows that increasing the extraction temperature from 30 to 140 °C resulted in a decrease in chlorophyll-a in the oil but had no significant (*p* < 0.05) effect on chlorophyll-b. Similar results were reported by Ayele et al. (2022), who reported that in all fractions of screw-pressed cassava leaves, the effect of increasing temperature was more pronounced on chlorophyll-a than on chlorophyll-b. This may be explained by the greater stability of chlorophyll-b with increasing temperature (Sánchez et al. 2014). In addition, a decrease in chlorophyll content at high temperatures has already been reported in hemp seed oils roasted at high temperatures, but no significant (*p* < 0.05) effect on carotenoids has been observed (Mansouri et al. 2023). However, the carotenoid content progressively increased with increasing extraction temperature.

**Table 3:** Results of color parameters (L*, a*, b*, C*_ab_, and h_ab_) of hemp seed oil extracted using a screw press at different temperatures

Temperature of extraction (°C)	color				
	L*	a*	b*	C*_ab_	h_ab_
Cold pressing (30)	45.31 ± 0.40b	-8.20 ± 0.25a	16.70 ± 0.19b	18.60 ± 0.47b	116.12 ± 0.01f
60	45.28 ± 0.11b	-7.63 ± 0.17a	17.40 ± 0.21c	18.99 ± 0.17bc	113.64 ± 0.01e
80	46.96 ± 0.54c	-7.33 ± 0.20ab	17.70 ± 0.30c	19.15 ± 0.32bc	112.46 ± 0.01d
100	45.73 ± 0.66bc	-6.96 ± 0.11b	17.44 ± 0.06c	18.77 ± 0.09bc	111.72 ± 0.01c
120	46.93 ± 0.21c	-7.08 ± 0.02b	19.31 ± 0.22d	20.56 ± 1.25c	110.10 ± 0.01b
140	45.52 ± 0.17b	-6.35 ± 0.10c	19.24 ± 0.99d	20.26 ± 0.71c	108.23 ± 0.01a
Soxhlet (*n*-hexane)	36.21 ± 0.09a	0.62 ± 0.03d	8.39 ± 0.14a	8.42 ± 0.14a	265.81 ± 0.29 g

Mean ± SD of three independent experiments (*n* = 3). abcdefg different letters indicate significant differences (*p* < 0.05) within columns

The color of the hemp seed oil samples was measured via a CIELAB color system. Table 3 shows the measurements of the parameters L*, a* and b* as well as other psychophysical color characteristics, including chromaticity (C*_ab_) and hue angle (h_ab_), of the extracted oils at different temperatures. The L* values ranged from 45.28 to 46.96. All extracted oils were positioned in the second quadrant of the CIELAB system, characterized by negative a* values (ranging from − 6.35 to -8.20, indicating a greenish hue) and positive b* values (ranging from 16.7 to 19.31, reflecting a yellowish hue).

The chroma values (C*_ab_) ranged from 18.60 to 20.56, whereas the hue angles (h_ab_) ranged from 108.23 to 116.12, confirming yellow‒green dominance. Notably, the high C*_ab_ values were influenced mainly by the b* values, as the a* values remained relatively low. Lower values for L*, b* and C*_ab_ (36.21, 8.39, and 8.42, respectively) but higher values for a* and h_ab_ (0.62 and 265.81, respectively) were observed for the Soxhlet-extracted oil.

The extraction temperature had a noticeable effect on the a* (green) and b* (yellow) color values of the oils. As the extraction temperature increased, the green (-a*) color decreased, whereas the yellow (+ b*) color increased. Similar trends for a* and b* color parameters have been reported for hemp seed oils roasted at high temperatures and extracted by screw presses (Mansouri et al. 2023). This color change was attributed mainly to the reduction in chlorophyll content, which gives the oil its green hue, and to the increase in carotenoid concentration, which is responsible for the yellow coloration. These results were consistent with the observations of Rabadán et al. (2018), who noted similar trends in the relationships between color parameters and the presence of chlorophyll and carotenoids in oils.

#### Fatty acid composition

Table 4 shows the effect of extraction temperature on the fatty acid profile of hemp seed oils. The composition of the oils obtained by the screw press is identical to those extracted by Soxhlet extraction with *n*-hexane. All the oils studied were characterized by a high content of polyunsaturated fatty acids (PUFAs), accounting for approximately 67%, including 53% omega-6 fatty acids and 14% omega-3 fatty acids, followed by monounsaturated fatty acids (MUFAs) (_~_18%) and saturated fatty acids (SFAs) (_~_14%). Among the fatty acids identified, linoleic acid comprises approximately half (_~_51%), followed by oleic acid (_~_18%), α-linolenic acid (_~_14%), palmitic acid (_~_10%), stearic acid (_~_3%) and γ-linolenic acid (_~_1%). The well-balanced omega-6/omega-3 ratio of approximately 3.60 was in line with the recommendations for a healthy diet.

**Table 4:** Results of fatty acids composition of hemp seed oil extracted using a screw press at different temperatures

Fatty acids (%)	Temperature of extraction (°C)						Soxhlet (*n*-hexane)
	Cold pressing (30)	60	80	100	120	140	
Palmitic acid (C16:0)	10.93 ± 0.85a	10.43 ± 0.83a	10.11 ± 0.36a	10.06 ± 0.59a	9.94 ± 0.66a	9.90 ± 0.72a	10.43 ± 0.59a
Stearic acid (C18:0)	2.70 ± 0.33a	2.78 ± 0.28a	3.01 ± 0.31a	2.80 ± 0.11a	2.83 ± 0.24a	2.80 ± 0.42a	3.23 ± 0.55a
Oleic acid (C18:1)	17.81 ± 0.25a	17.81 ± 0.28a	18.89 ± 0.79a	18.76 ± 0.28a	18.11 ± 0.16a	18.65 ± 0.23a	18.50 ± 0.66a
Linoleic acid (C18:2n6)	51.61 ± 0.40a	52.14 ± 0.37a	50.91 ± 0.78a	51.27 ± 0.27a	51.77 ± 0.94a	51.68 ± 0.46a	51.32 ± 0.33a
γ-Linolenic acid (C18:3n6)	1.06 ± 0.01a	1.05 ± 0.06a	1.05 ± 0.01a	1.02 ± 0.01a	1.04 ± 0.05a	1.05 ± 0.01a	1.05 ± 0.01a
α-Linolenic acid (C18:3n3)	14.88 ± 0.34a	14.68 ± 0.45a	14.85 ± 0.50a	14.87 ± 0.20a	15.05 ± 0.19a	14.66 ± 0.56a	14.52 ± 0.54a
Arachidic acid (C20:0)	0.45 ± 0.04a	0.44 ± 0.09a	0.56 ± 0.06b	0.63 ± 0.05b	0.62 ± 0.03b	0.60 ± 0.14b	0.39 ± 0.01a
Eicosenoic acid (C20:1)	0.18 ± 0.01a	0.20 ± 0.02a	0.17 ± 0.02a	0.18 ± 0.01a	0.18 ± 0.01a	0.18 ± 0.02a	0.16 ± 0.02a
Behenic acid (C22:0)	0.24 ± 0.01a	0.32 ± 0.03ba	0.32 ± 0.01b	0.27 ± 0.02a	0.32 ± 0.03b	0.34 ± 0.06b	0.28 ± 0.03ab
Lignoceric acid (C24:0)	0.13 ± 0.01a	0.15 ± 0.00a	0.16 ± 0.01a	0.16 ± 0.01a	0.16 ± 0.01a	0.15 ± 0.01a	0.15 ± 0.01a
SFA	14.91 ± 1.04a	14.57 ± 1.12a	14.70 ± 0.46a	14.33 ± 0.79a	14.48 ± 0.96a	14.39 ± 0.83a	14.85 ± 0.18a
MUFA	17.99 ± 0.27a	18.01 ± 0.26a	19.05 ± 0.40b	18.94 ± 0.26b	18.29 ± 0.15a	18.83 ± 0.22b	18.66 ± 0.67b
PUFA	67.56 ± 0.73a	67.87 ± 0.77a	66.8 ± 0.94a	67.16 ± 0.48a	67.85 ± 1.08a	67.38 ± 1.11a	66.88 ± 0.87a
UFA	85.54 ± 1.00a	85.87 ± 1.03a	85.85 ± 0.40a	86.09 ± 0.74a	86.14 ± 0.93a	86.21 ± 1.01a	85.54 ± 0.80a
Omega-6 (n-6)	52.68 ± 0.39a	53.19 ± 0.31a	51.95 ± 0.80a	52.29 ± 0.28a	52.81 ± 0.89a	52.73 ± 0.45a	52.37 ± 0.32a
Omega-3 (n-3)	14.88 ± 0.34a	14.68 ± 0.45a	14.85 ± 0.50a	14.87 ± 0.2a	15.05 ± 0.19a	14.66 ± 0.56a	14.52 ± 0.54a
n-6/n-3	3.54 ± 0.05a	3.62 ± 0.09a	3.50 ± 0.17a	3.52 ± 0.13a	3.51 ± 0.10a	3.60 ± 0.17a	3.61 ± 0.11a

Mean ± SD of three independent experiments (*n* = 3). abc different letters indicate significant differences (*p* < 0.05) within rows, ns, non-significative. MUFA, Monounsaturated fatty acids; PUFA, Polyunsaturated fatty acids; SFA, Saturated fatty acids; UFA, Unsaturated fatty acids

The fatty acid profiles of oils extracted at different temperatures agreed with the results of previous studies (Abdollahi et al. 2020; Galasso et al. 2016). Furthermore, oils obtained from roasted and unroasted hemp seeds presented similar profiles, regardless of the ecotype studied (Mansouri et al. 2023). However, some studies have reported higher proportions of linoleic acid (up to 56%), α-linolenic acid (up to 18%) and γ-linolenic acid (up to 3%), with lower proportions of oleic acid (_~_10%) (Golimowski et al. 2022; Occhiuto et al. 2022). These variations could be explained by genetic differences or climatic conditions, as confirmed by several studies showing that the fatty acid composition of hemp oils is strongly influenced by these factors (Irakli et al. 2019; Taaifi et al. 2021).

Although high PUFA contents give oils beneficial health properties, they also increase their susceptibility to oxidation (Rabadán et al. 2018). However, in this study, the use of a screw press at extraction temperatures of 30–140 °C caused neither oxidation nor isomerization of the fatty acids. This may be attributed to the increased content of antioxidant compounds, notably tocopherols, in oils extracted at high temperatures, which play a protective role against free radical formation and fatty acid degradation (Farinon et al. 2020).

Similar results were reported by Golimowski et al. (2022), who found no significant difference in the fatty acid profile between cold-extracted and hot-extracted hemp seed oils, regardless of variety. Similarly, the work of Rabadán et al. (2018) on pistachio and walnut oils indicated that the extraction temperature did not affect their fatty acid profile. Mansouri et al. (2023) demonstrated that even a severe roasting process (163 °C for 15 min) had no negative effect on the PUFAs of hemp seed oils. Only minor variations were observed for oleic acid and palmitic acid.

#### Oxidative stability and quality indices

The oxidation stability provides information on the oil quality, since it refers to the sensitivity of the oil to oxidation. The degree of unsaturation of fatty acids is the main factor affecting oil stability. The more unsaturated fatty acids an oil contains, the more sensitive it is to oxidation (Farinon et al. 2020). Several compounds present in the oil, such as free fatty acids, metals and chlorophylls, have oxidation-promoting effects (Farinon et al. 2020). On the other hand, antioxidant compounds increase the oxidative stability of oils. The presence of antioxidants increases the oxidation induction time or slows the oxidation rate (Allay et al. 2024a). The tocopherol family is the main antioxidant in hemp seed oil, primarily due to its significantly higher concentration compared to other antioxidants. The antioxidant activity of tocopherols depends on the amount present in the oil, among other factors. Polyphenols are also antioxidants, but their low quantity in the oil makes them less effective than tocopherols (Farinon et al. 2020).

**Table 5:** Results of peroxide value (PV), percentage free fatty acid (FFA) oxidation stability index (OSI) and specific extinction coefficient (conjugated diene (K232) and triene (K270)) of hemp seed oil extracted using a screw press at different temperatures

Temperature of extraction (°C)	Specific extinction coefficients		FFA (%)	PV (meq O_2_/Kg)	OSI (hours)
	Conjugated diene (K232)	Conjugated triene (K270)			
Cold pressing (30)	1.54 ± 0.01a	0.38 ± 0.02a	0.081 ± 0.003a	6.36 ± 0.30a	11.13 ± 0.13a
60	1.57 ± 0.02a	0.38 ± 0.01a	0.083 ± 0.002a	6.46 ± 0.51a	11.10 ± 0.21a
80	1.75 ± 0.01b	0.40 ± 0.02a	0.083 ± 0.002a	6.40 ± 0.57a	11.58 ± 0.17a
100	1.75 ± 0.01b	0.40 ± 0.01a	0.094 ± 0.004b	8.30 ± 0.47b	11.30 ± 0.28a
120	2.03 ± 0.04c	0.40 ± 0.01a	0.098 ± 0.005b	8.53 ± 0.84b	10.42 ± 0.15b
140	2.06 ± 0.02c	0.41 ± 0.01a	0.100 ± 0.003b	13.86 ± 0.37c	10.29 ± 0.11b
Soxhlet (*n*-hexane)	1.82 ± 0.16bc	0.55 ± 0.02b	1.95 ± 0.05c	20.80 ± 2.77d	13.97 ± 0.62c

Mean ± SD of three independent experiments (*n* = 3). abc different letters indicate significant differences (*p* < 0.05) within column

Table 5 shows the results for oxidative stability, which was assessed by determining the induction time via the Rancimat accelerated aging method. The induction time ranged from 10.29 h (oil extracted at 140 °C) to 11.58 h (oil extracted at 80 °C). The induction time decreased slightly with increasing temperature, indicating that oil extracted at high temperatures was sensitive to oxidation. This may be due to the increase in primary oxidation products (peroxides and hydroperoxides). In addition, the overall oxidative stability of the oils extracted by the screw press was lower than that extracted with *n*-hexane at 13.97 h. Although high temperatures of up to 140 °C were used in our study, the OSI values obtained are higher than those reported by Tura et al. (2023), which ranged from 3.37 to 6.72 h. In contrast, Mansouri et al. (2023) reported an OSI value of 11.83 h, comparable to our results. This difference could be explained by the fatty acid composition, in particular the PUFA content, known for its sensitivity to oxidation. The oils studied by Tura et al. (2023) had a higher PUFA content than those analyzed in our study and in that of Mansouri et al. (2023) which justifies their lower oxidative stability.

To illustrate the effect of extraction temperature on oil quality, various chemical analysis methods were used, such as PV, FFA and specific extinctions (K232 and K270). The PV values ranged from 6.36 meq O_2_/kg for oil extracted at a temperature of 30 °C to 13.86 meq O_2_/kg for oil extracted at a temperature of 140 °C (meq O_2_/kg), but analysis of variance revealed that there was no significant (*p* < 0.05) difference between oils extracted at temperatures of 30, 60 and 80 °C until the values became significant (*p* < 0.05) at temperatures of 100 °C. Furthermore, the FFA values ranged from 0.081% (oil extracted at 30 °C) to 0.100% (oil extracted at 160 °C), but similar to the PV, the values were significantly different at temperatures above 100 °C (*p* < 0.05) (Table 5).

In addition, the specific extinction coefficients K232 and K270 are also able to describe the primary (peroxides) and secondary (aldehydes, ketones) oxidation products present in the oil samples. As shown in Table 5, the K232 values ranged from 1.54 to 2.06, with values differing significantly between oils extracted at different temperatures (*p* < 0.05). However, no significant (*p* < 0.05) difference was observed in the K270 values, which ranged from 0.38 to 0.41. Furthermore, the oil quality indices obtained in our study are in close agreement with those reported in the literature (Muangrat and Kaikonjanat 2025; Tura et al. 2023).

Furthermore, the quality of oil obtained by pressing at different temperatures was superior to that obtained by Soxhlet, with PV of 20.80 meq O_2_/kg, FFA of 1.95%, K232 of 1.82 and K270 of 0.55. This could be explained by the long process time (6 h) (Rezvankhah et al. 2019).

Table 5 shows that the %FFA values increased with increasing temperature. Similar results were reported by Aladić et al. (2014) that the %FFA of hemp seed oils extracted via a screw press increased significantly as the temperature increased from 2.49% at 60 °C to 2.64% at 100 °C. According to Aladić et al. (2014), increasing the temperature increased the FFA content via enzymatic hydrolysis of glycerides with the release of fatty acids. In addition, the press temperature had a significant (*p* < 0.05) effect on the PV and K232 of the extracted oils, possibly because of the increase in FFA, which served as the primary substrate for oxidation reactions. Fatty acids lose a proton, forming free radicals, which react with oxygen to form unstable peroxide radicals that subsequently react with other fatty acid molecules to generate hydroperoxides (Allay et al. 2024a). However, no significant (*p* < 0.05) effect was observed on K270. In our study, although high extraction temperatures of up to 140 °C were used, the oil outlet temperatures were well below 62 °C. This may be the reason for the absence of a significant difference in K270. Because λ 270 measures of secondary oxidation products, high temperatures and long oil storage times are required for the evolution of these oxidation compounds.

### Chemometric analysis

Principal component analysis (PCA) was used to assess the effect of the screw press extraction temperature on the properties of the hemp seed oils. This statistical method reduces the dimensionality of the data while preserving most of the variance, thus facilitating the identification of the most influential factors.

Prior to analysis, the data were tested for goodness-of-fit via the Kaiser Meyer Olkin (KMO) test and Bartlett’s test, taking all the parameters into account. The variables selected for good data fit were OY, oil temperature, TPC, total tocopherol, total chlorophyll, total carotenoid, SFA, UFA, omega-3, omega-6, PV, FFA, K232 and OSI, with a KMO score of 0.718, suggesting that the correlation between these variables was strong enough to justify PCA. In addition, Bartlett’s test was highly significant (*p* < 0.001), confirming that the correlation matrix was suitable for this analysis.

**Fig. 3:**
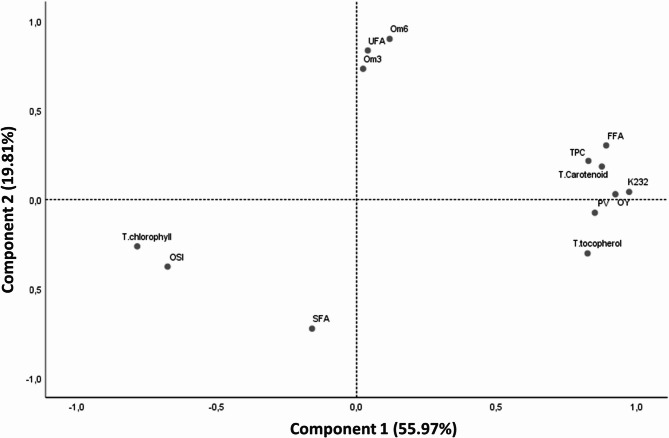
Principal component analysis (PCA) bi-plot of hemp seed oil parameters, oil yield (OY), oil temperature (T°C.Oil), total phenolic content (TPC), total tocopherol (T.Tocopherol), total chlorophyll (T.Chlorophyll), total carotenoid (T.Carotenoid), saturated fatty acids (SFA), unsaturated fatty acids (UFA), omega-3, omega-6, peroxide value (PV), free fatty acid (FFA), conjugated diene (K232) and oxidative stability index (OSI) obtained by mechanical extraction at different temperatures (30, 60, 80,100, 120 et 140 °C)

PCA was used to group the extracted oils according to their similarity in terms of yield, oil temperature, chemical composition (TPC, total tocopherol, total chlorophyll, total carotenoid, and fatty acids such as SFA, UFA, omega-3 and omega-6) and quality (PV, FFA, K232 and OSI). The results (Fig. 3) show that the first two principal components (CP1 and CP2) together explained 75.78% of the total variance, with 55.97% for CP1 and 19.81% for CP2. CP1 was positively correlated with temperature-influenced parameters such as OY, oil temperature, total tocopherol content, TPC, total carotenoid content and quality indices (PV, FFA and K232) but negatively correlated with total chlorophyll content and the OSI. CP2, on the other hand, reflected variations in fatty acid composition, showing a positive correlation with UFAs, omega-3 fatty acids and omega-6 fatty acids and a negative correlation with SFAs.

**Fig. 4:**
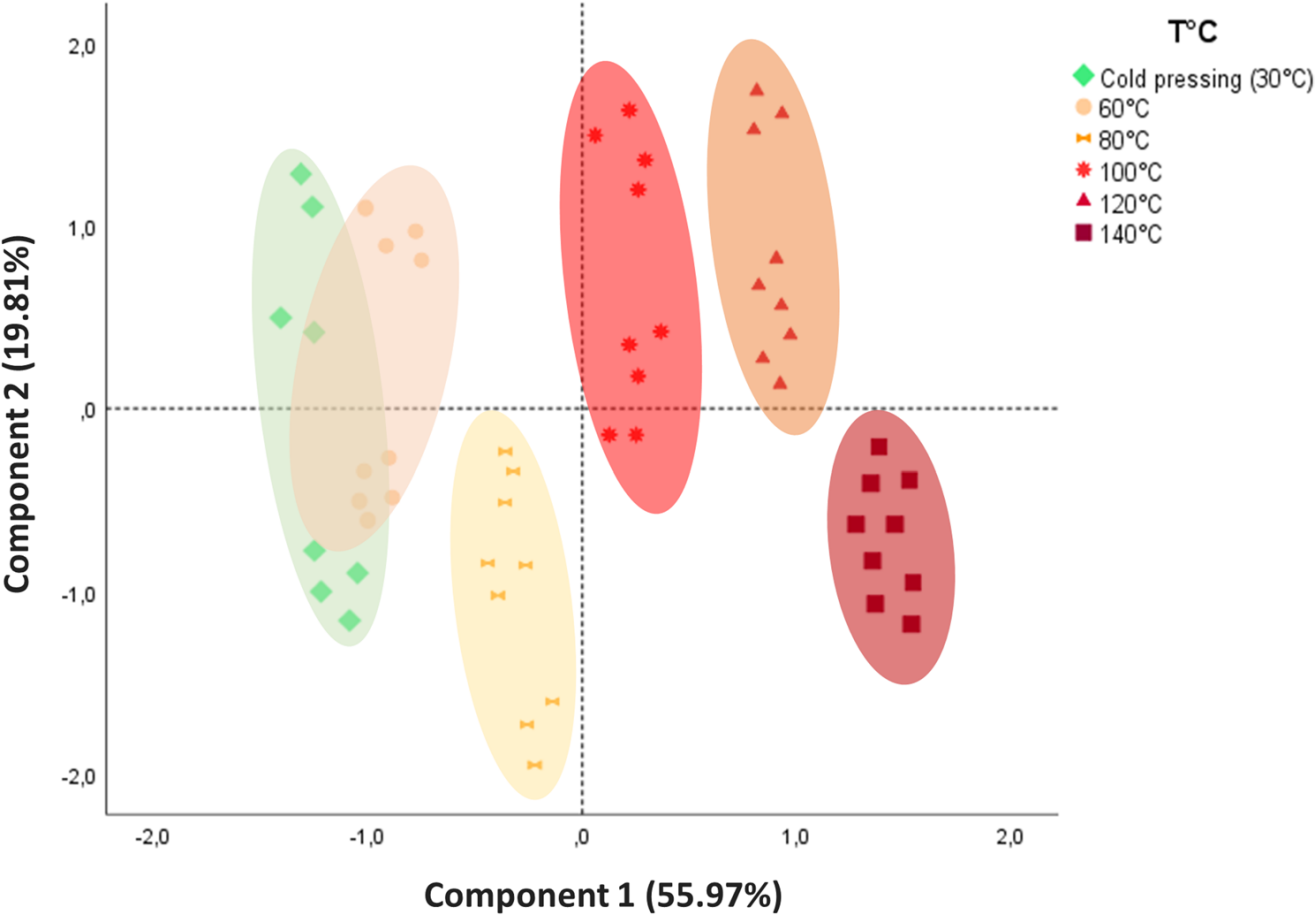
Principal component analysis (PCA) biplot of hemp seed oils obtained by extraction mechanical at different temperatures (30, 60, 80,100, 120 et 140 °C)

The biplot (Fig. 4) clearly illustrates the significant impact of extraction temperature on hemp oil properties. At lower extraction temperatures (30–80 °C), oils were grouped into a quadrant associated with higher total chlorophyll content and better OSI, as evidenced by lower PV, FFA and K232. This suggests that moderate temperatures help preserve essential bioactive compounds while minimizing oxidative degradation. In contrast, higher extraction temperatures (100, 120 and 140 °C) were associated with increased OY and higher levels of total tocopherols, TPC and total carotenoids. However, these benefits were achieved at the cost of reduced oil quality, as indicated by significant increases in PV, FFA and K232. The degradation of chlorophyll pigments at elevated temperatures was particularly noteworthy, as their concentrations fell sharply, suggesting substantial thermal degradation. Furthermore, oils extracted at 100 and 120 °C were found to be richer in unsaturated fatty acids (UFAs), particularly omega-6 and omega-3, while oils obtained at lower temperatures, particularly 80 °C, contained a higher proportion of saturated fatty acids (SFAs). This variation in fatty acid composition highlights the influence of extraction temperature on oil properties.

These results demonstrate that the extraction temperature of the screw press was a determining factor in the yield, composition and quality of hemp seed oils. Although high temperatures increase OY, they also lead to quality degradation, with increases in the PV, FFA and K232, as well as a decrease in the oxidative stability. Therefore, to produce high-quality oil, it is essential to optimize the extraction temperature according to priority, whether by maximizing yield or preserving bioactive properties and stability.

## Conclusion

Pressing temperature played a decisive role in the extraction of oil from hemp seeds using a screw press. An increase in temperature significantly improved OY, which reached a maximum of 21.82% at 100 °C, an increase of around 4% compared to cold pressing (30 °C). In addition, a relatively high extraction temperature favored the extraction of bioactive compounds, in particular tocopherols, which helped protect the oil against oxidation. In addition, the fatty acid profile remained stable, whatever the temperature applied. However, temperatures above 80 °C compromised the quality of the oil, as evidenced by increases in PV, FFA and K232 indices, leading to a decrease in its oxidation stability.

Although the temperature improved OY, it also had negative effects on its quality. To optimize the process while preserving product properties, it is essential to explore innovative solutions, including the integration of combined technologies such as ultrasonic or microwave-assisted extraction. Optimization of other extraction parameters, such as screw rotation speed and nozzle diameter, could also enhance process efficiency. Finally, beyond technical consideration, a global approach that considers the economic and environmental dimensions of this method is necessary to ensure its viability and sustainability.

## Data Availability

No datasets were generated or analysed during the current study.
